# We Grow

**Published:** 1869-07

**Authors:** 


### The Bistoury.

ELMIRA, N. Y., JULY, 1869.

### EDITORIAL.

### WE GROW.

Yes, we are in a flourishing condition. Our pocket-book is made plethoric with the fifty cent "shin-plasters" of our friends—therefore thrive we, which accounts for our printing the BISTOURY upon better paper, using better materials, and furnishing better articles. It is sort of *jolly* to write for money—some-how-or-other it is easier—one finds a subject more readily, and can do it justice after it is found. We only wonder why we didn't learn long ago, that we could secure more subscribers for our journal at fifty cents apiece, than for *nothing*. But we have made the discovery finally, and now mean to profit by it. How we grow, to be sure! From a circulation of three thousand, to that of more than *twenty thousand!* If so much can be accomplished in three years, how much will we grow in the *next* three? We hav'nt time to solve the problem now, but will assure the many patrons of the BISTOURY that we thank them heartily for responding so handsomely to our subscription price. We can now write to some purpose, and will continue to improve and enlarge our journal as fast as the subscriptions of our friends will allow us to. Give us the means to defray the expenses of publishing this journal, and we will give you the best and cheapest family magazine in America.

Our list of physicians, who take the BISTOURY, has nearly doubled since our last issue. An evidence that our "Medical Items and News" is duly appreciated by them. We offer, for fifty cents a year, that which will cost the physician more than twenty-five dollars to procure. All that is transpiring in the medical world, and reported through the numerous medical journals, we collect and condense, giving an epitome of all that is new and valuable to the practitioner of medicine.

In short, we've got a good journal, *we* know it, and are determined that the *people* shall acknowledge it. You can no more do without the BISTOURY than you can without your daily paper. Therefore subscribe for it—read it—be informed—be happy.

